# A three-by-three cluster crossover trial of post-acute care patients with fracture

**DOI:** 10.1093/geroni/igaf122.3086

**Published:** 2025-12-31

**Authors:** Thomas Travison, Cathleen Colon-Emeric, Mark Toles, Andrew Zullo, Stephanie Kissam, Sarah Berry

**Affiliations:** Hebrew SeniorLife & Harvard Medical School, Hebrew SeniorLife, Massachusetts, United States; Duke University School of Medicine, Durham, North Carolina, United States; The University of North Carolina at Chapel Hill, Chapel Hill, North Carolina, United States; Brown University, Providence, Rhode Island, United States; American Health Care Association, Washington, District of Columbia, United States; Hebrew SeniorLife & Harvard Medical School, Hebrew SeniorLife, Massachusetts, United States

## Abstract

Later-life fractures are a leading cause of disability. Medication optimization improves outcomes, but no studies have compared models to optimize medications in skilled nursing facilities (SNFs). To address this gap, we designed a pragmatic three-by-three cluster-crossover trial to evaluate three post-acute care models: 1) a Bone Health model providing osteoporosis management recommendations; 2) a Deprescribing model targeting medications that increase fall and fracture risk; and 3) a Combined Care model incorporating both models. In all models, a remote nurse consultant collaborates with an interprofessional team to provide personalized medication recommendations to SNF providers. Forty-two SNFs will be randomized to deliver each model for six months in a randomized sequence such that all SNFs eventually deploy all three models. Approximately 3,780 patients with fracture will receive the intervention while in SNF care. 126 control facilities will be matched to the intervention facilities to compare outcomes. The primary endpoint, injurious falls, will be identified through Medicare claims linked with electronic health record data in the Long-Term Care Data Cooperative, for up to two years post-discharge. Secondary endpoints include fall risk-increasing medication exposure, falls self-efficacy, pain, and sleep quality, measured over 90 days post-discharge. This study is one of few cluster-crossover trials comparing more than two interventions. The presentation will highlight key design features, statistical power computations and considerations, planned statistical analyses, and the trial progress, offering insights for researchers interested in applying this unique cluster-crossover design.

